# Deficient spontaneous *in vitro* apoptosis and increased tmTNF reverse signaling-induced apoptosis of monocytes predict suboptimal therapeutic response of rheumatoid arthritis to TNF inhibition

**DOI:** 10.1186/ar4416

**Published:** 2013-12-20

**Authors:** Undine Meusch, Maria Klingner, Christoph Baerwald, Manuela Rossol, Ulf Wagner

**Affiliations:** 1 Division of Rheumatology, Department of Internal Medicine, University of Leipzig, Liebigstr.20, 04103 Leipzig, Germany

## Abstract

**Introduction:**

*In vitro* apoptosis of peripheral monocytes in rheumatoid arthritis (RA) is disturbed and influenced by cytokine production and transmembrane TNF (tmTNF) reverse signaling. The goal of the study was the analysis of the predictive value of the rate of *in vitro* apoptosis for the therapeutic response to anti-TNF treatment.

**Methods:**

Spontaneous and tmTNF reverse signaling-induced apoptosis were determined *in vitro* in monocytes from 20 RA patients prior to initiation of therapeutic TNF inhibition with etanercept, and the subsequent clinical response was monitored.

**Results:**

Spontaneous *in vitro* apoptosis was significantly reduced in RA patients compared to controls. Deficiency in spontaneous apoptosis was associated with an insufficient therapeutic response according to the European League Against Rheumatism (EULAR) response criteria and less reduction of the disease activity determined by disease activity score (DAS) 28. High susceptibility to reverse signaling-induced apoptosis was also associated with less efficient reduction in the DAS28. Of note, a strong negative correlation between the two apoptotic parameters was discernible, possibly indicative of two pathogenetically relevant processes counter-regulating each other.

tmTNF reverse signaling induced *in vitro* production of soluble IL1-RI and IL-1RII only in monocytes not deficient in spontaneous apoptosis, and the levels of soluble IL1-RII were found to be predictive of a good clinical response to Etanercept.

**Conclusion:**

Although tmTNF reverse signaling is able to induce apoptosis of RA monocytes *in vitro*, this process appears to occur *in vitro* preferentially in patients with suboptimal therapeutic response. Resistance to spontaneous *in vitro* apoptosis, in contrast, is a predictor of insufficient response to treatment.

## Introduction

Rheumatoid arthritis (RA) is a systemic autoimmune disease primarily affecting diarthrodial joints of hand and feet, but clinical patterns vary widely. Inflammatory synovitis and subsequent joint destruction in RA is to a large extent driven by the monocytic cytokines TNF, IL-6 and IL-1β. Therapeutic cytokine inhibition, and in particular inhibition of TNF, is highly effective in a high percentage of patients. The precise mode of action of therapeutic TNF blockade is not clear, and several mechanisms have been suggested. Neutralization of soluble as well as membrane-anchored TNF is believed to be the primary mechanism behind the clinical efficacy in preventing joint destruction, but as an alternative mode of action of TNF-blocking agents, outside-to-inside signals through transmembrane TNF-alpha has also been suggested [1,2]. The latter is likely to contribute to certain anti-TNF effects exerted on immune cells such as migratory inhibition [3] and increased apoptosis [4].

Recently, our group was able to identify profound differences in monocyte apoptosis between RA patients and healthy donors. Monocytes from healthy controls undergo spontaneous apoptosis (SIA) *in vitro* at considerable rates during incubation over 16 hours. In monocytes from RA patients, SIA is significantly reduced [5]. Deficient spontaneous *in vitro* apoptosis has also been reported by other groups for peripheral monocytes from patients with systemic juvenile idiopathic arthritis [6] and for monocytic cells from the rheumatoid synovium [7,8].

In addition to deficient SIA, monocytes from RA patients are also abnormally susceptible to *in vitro* apoptosis induced by incubation with TNF-blocking agents. The *in vitro* mode of action of those agents is ligation-triggered reverse signaling (RS) of the transmembrane TNF molecule (tmTNF) [9], which induces the *in vitro* apoptosis (tmTNF reverse signaling-induced apoptosis, tmTNF RSA) [5]. One mechanism leading to tmTNF RSA is the inhibition of the excessive IL-1β secretion of RA monocytes via tmTNF RS [5].

Several members of the IL-1 family of cytokines and receptors are involved in the pathogenesis and the regulation of disease activity in RA. IL-1β is overexpressed in arthritic joints, and therapeutic inhibition of IL-1β with anakinra is an established treatment option. IL-1β (as well as IL-1α) binds to the transmembrane ligand-binding chain of the IL-1 receptor (termed IL-1R type I) as well as to the IL-1 receptor type II (IL-1RII), which lacks a cytoplasmic domain and functions as a decoy receptor for IL-1β [10,11]. Both receptors can be released from the cell surface in a soluble form as IL-1sRI and IL-1sRII, but increased neutralization capacity had been shown for IL-1sRI [12].

In the present study, we have investigated both spontaneous *in vitro* apoptosis and consequences of tmTNF RS in a cohort of RA patients, treated subsequently with the TNF inhibitor etanercept. The results show that the RA-specific, abnormal *in vitro* apoptosis of RA patients is a predictor of their subsequent clinical response.

## Methods

### Patients and study design

The design of the clinical study had been approved by the ethics committee of the University of Leipzig, and informed consent was obtained from each patient before study enrollment. A total of 33 patients with RA according to the revised criteria of the American College of Rheumatology [13] was recruited. None of the patients had previously been treated with TNF inhibitors. Ten healthy donors served as controls. For the initial pre-study cohort, 13 patients with a mean age of 64 years and mean disease duration of 16 years were recruited: 11 of these patients (78%) were seropositive for rheumatoid factor IgM (RF IgM), and 9 patients (75%) had anti-cyclic citrullinated peptid (anti-CCP) antibodies.

In the longitudinal clinical study, 20 patients were initiated on treatment with etanercept due to clinical requirements and clinical and laboratory parameters of disease activity were monitored at baseline and throughout the study. In this cohort, the mean age was 53 years, and the mean disease duration was 4 years: 65% of the patients were RF IgM-seropositive, and 80% had anti-CCP antibodies. At baseline, 80% (16 patients) were treated with conventional disease-modifying anti-rheumatic drugs (DMARDs), given either as monotherapy or in combination, and 20% received low-dose glucocorticoids only. All patients were treated with non-steroidal anti-inflammatory drugs (NSAIDs) for symptomatic relief. All concomitant medication remained unchanged upon initiation of etanercept treatment and throughout the study.

### Monocyte isolation, cell culture and cell stimulation

Monocytes from peripheral blood were separated as previously described [9]: 2 × 10^5^ monocytes per 200 μl were incubated in Roswell Park Memorial Institute medium (RPMI) 1640 supplemented with 5% human AB serum (heat inactivated). Stimulation of cells was carried out either with 100 mg/ml rituximab (Roche, Basel, Switzerland) as an IgG control or with the soluble TNFR2:Ig construct etanercept (Pfizer, New York, NY, USA) for 16 hours.

### Apoptosis detection

Staining of apoptotic and necrotic cells was performed by using 10 μl allophycocyanin-labeled Annnexin V (Southern Biotechnology, Birmingham, AL, USA) and 50 μg/ml propidium iodide (IP), respectively. Fluorescence was measured on the FACSCalibur system, and the results were analyzed using FlowJo software (Tree Star, Inc, Ashland, OR, USA).

### Cytokine detection

Cytokine levels in cell culture supernatants were measured with cytometric bead arrays (BD biosciences, Franklin Lakes, NJ, USA) according to the manufacturer's protocol.

### Statistics

For statistical analysis, graphPad Prism software (GraphPad Software, La Jolla, CA, USA) was used. Prior to all comparisons, a normality test was performed. To assess statistical significance, Student’s *t*-test (normal distribution of data) or the Mann–Whitney rank sum test (unequal distribution of data) was used. Correlation between two parameters was analyzed with Pearson’s product–moment correlation.

## Results

### The soluble TNFR2: Ig construct etanercept triggers tmTNF RSA in RA monocytes

**Figure 1:**
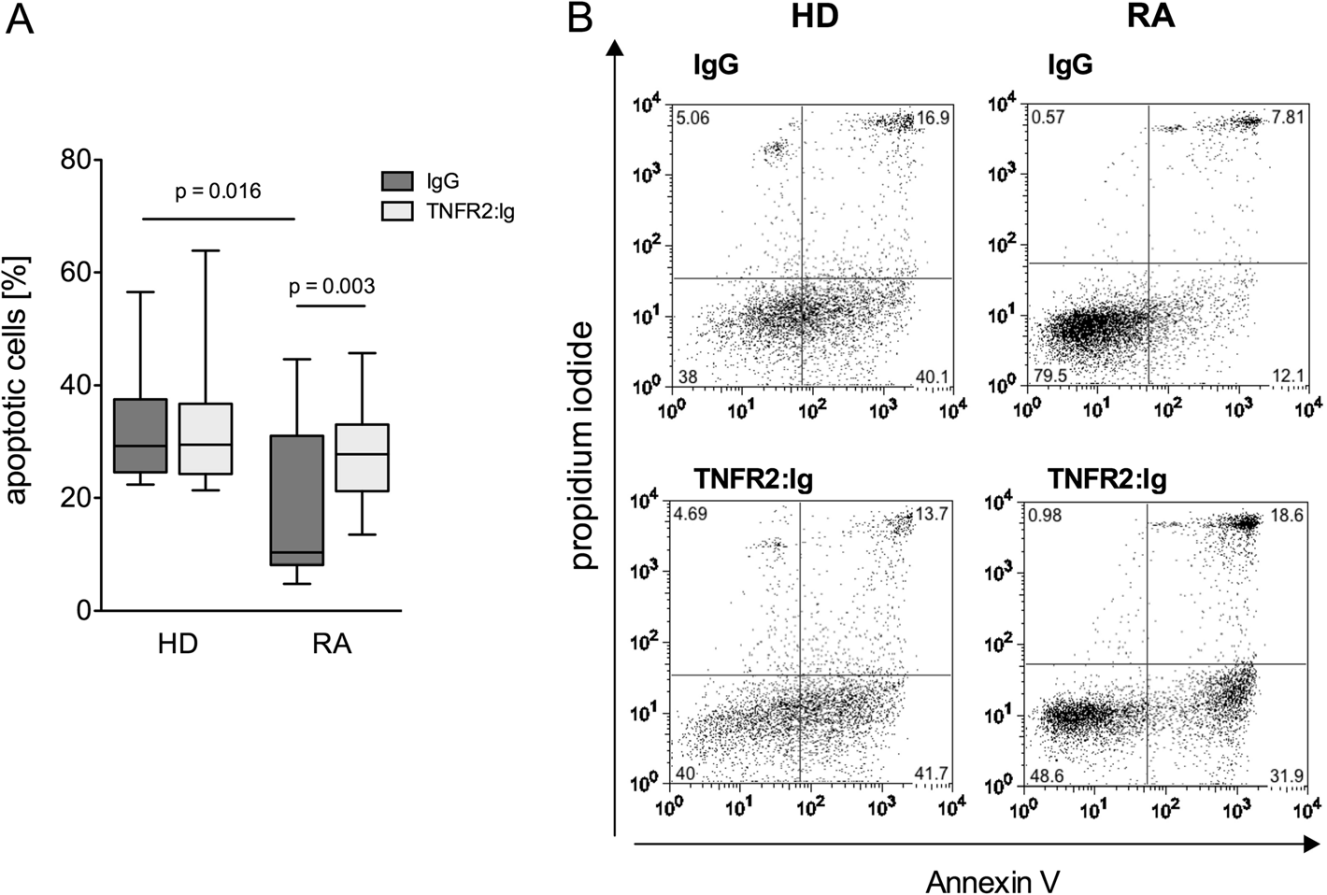
**TNFR2:Ig triggered reverse signaling via transmembrane (tm)TNF induces apoptosis in monocytes from rheumatoid arthritis (RA) patients but not from healthy controls. (A)** Monocytes from the peripheral blood of healthy donors (HD) and from RA patients (RA) were cultured with IgG or TNFR2:Ig for 16 hours. Box plot depicts the rate of spontaneous apoptosis *in vitro* (SIA) cultures with IgG (dark gray bars) and of tmTNF reverse signaling-induced apoptosis (tmTNF RSA) in the presence of TNFR2:Ig (light gray bars) in healthy controls (HD) (n = 10) and RA patients (RA) (n = 13). Significant differences are as indicated. **(B)** Representative dot plots of annexin V- and propidium iodide-stained monocytes from a healthy control (left panels) and from one RA patient (right panels) after incubation with IgG or TNFR2:Ig for 16 hours. Upper panels show spontaneous apoptosis *in vitro* (SIA) (IgG), lower panels tmTNF reverse signaling induced apoptosis (tmTNF RSA) (TNFR2:Ig).

We have shown previously, that ligation of tmTNF by anti-TNF antibodies induces apoptosis in RA monocytes through triggering tmTNF RS [5]. In addition, this study found a decreased rate of spontaneous *in vitro* apoptosis in monocytes from RA patients compared to healthy controls. For the present study, an initial pre-study investigation confirmed the lower rate of spontaneous monocyte apoptosis in RA patients compared to healthy controls (RA: 18.92 ± 13.56% versus healthy donors (HD): 32.44 ± 10.52%, *P* = 0.016, Figure 1A,B). Triggering of tmTNF RS by ligation with the soluble TNFR2:Ig construct etanercept yielded rates of tmTNF RSA that were similar to those obtained with the anti-TNF antibody in our previous study [5]. The median rate of tmTNF RSA in RA patients was 1.6-fold higher than the spontaneous apoptosis (tmTNF RSA: 28.56 ± 9.1% versus SIA: 18.92 ± 13.56%, n = 13, *P* = 0.003, Figure 1A), whereas RS did not influence the rate of *in vitro* apoptosis in healthy controls (32.64 ± 12.20 versus 32.44 ± 10.52, n = 10, not significant, Figure 1A). As etanercept was used in the treatment of the patients in the study, it was also chosen as the TNF-blocking agent for the *in vitro* experiments in the longitudinal study presented here.

### Deficient SIA is a predictor of insufficient therapeutic response

Clinical characteristics of the main study cohort and parameters of disease activity at baseline and after 12 weeks of TNF blockade are summarized in Table 1. After 12 weeks, 50% of the patients achieved a good clinical response according to the European League Against Rheumatism (EULAR) response criteria for RA [14], whereas 50% achieved only a moderate response or no response.

**Table 1:** Baseline and week-12 characteristics in patients

	**All patients**	**Baseline**			**Week 12**	
		**Responder (week 12)**	**Nonresponder (week 12)**	**All patients**	**Responder**	**Nonresponder**
Number	20	10	10	20	10	10
TJC of 28 joints	8.25 (±7.2)	9.20 (±8.7)	7.30 (±5.8)	3.16 (±3.3)	2.20 (±3,2)	4.22 (±3.3)
SJC of 28 joints	5.45 (±3.7)	5.90 (±3.1)	5.00 (±4.3)	1.79 (±2.7)	0.70 (±1.9)	3.00 (±3.1)
VAS general disease activity	47.8 (±20.4)	52.6 (±19.3)	42.9 (±21.4)	27.6 (±18.6)	21.6 (±16.9)	34.2 (±19.1)
ESR, mm/h	23.7 (±13.6)	19.1 (±10.5)	28.3 (±15.3)	17.8 (±14.2)	11.1 (±7.3)	25.1 (±16.7)
C reactive protein, mg/dl	7.22 (±6.5)	6.65 (±4.9)	7.79 (±8.0)	5.40 (±8.9)	2.71 (±3.1)	8.3 (±12.3)
DAS28	4.76 (±1.2)	4.82 (±1.2)	4.7 (±1.2)	3.33 (±1.4)	2.50 (±1.1)	4.17 (±1.2)
Age, years	53.65 (±9.4)	53.5 (±10.7)	53.8 (±8.5)	-	-	-
Sex, female, %	70	60	80	-	-	-
Disease duration, years	4.35	4.3	4.4	-	-	-
Rheumatoid factor, +/-, %	65/35	70/30	60/40	-	-	-
Anti CCP, +/-, %	80/20	80/20	80/20	-	-	

TJC, tender joint count; SJC, swollen joint count; VAS, visual analog scale; ESR, erythrocyte sedimentation rate; DAS28, disease activity score in 28 joints; CCP, cyclic citrullinated peptide.

**Figure 2:**
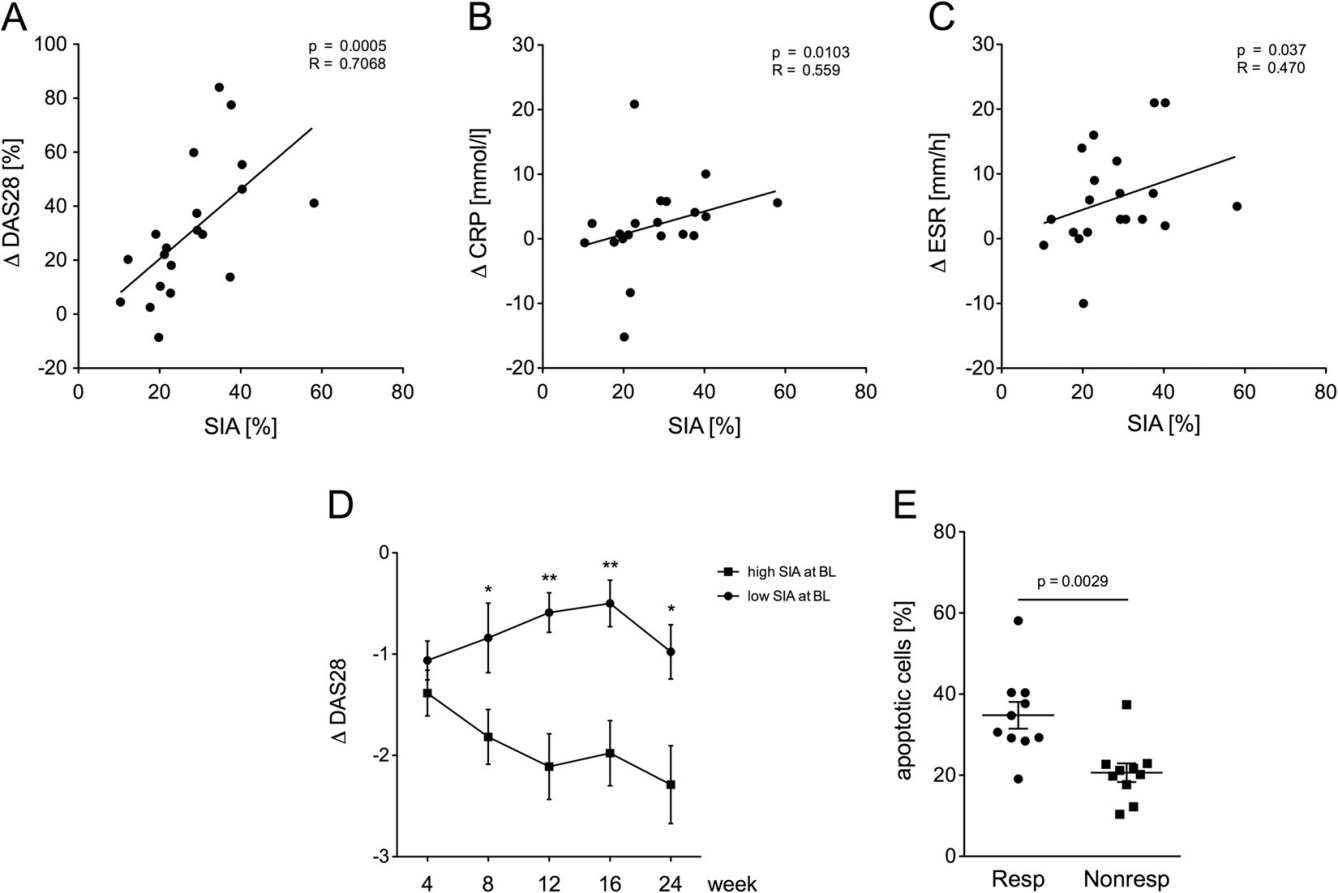
**Spontaneous *in vitro *apoptosis of monocytes correlates with change in disease activity after 12 weeks. (A-C)** Spontaneous apoptosis of monocytes from rheumatoid arthritis (RA) patients was measured at baseline and the change in disease activity score (DAS)28, C-reactive protein (CRP) and erythrocyte sedimentation rate (ESR) levels from baseline and to week 12 after starting treatment with etanercept for these patients was calculated. Dot plots depict the rate of spontaneous *in vitro* apoptosis (SIA) in relation to the change (Δ) in DAS28 **(A)**, CRP **(B)** and ESR **(C)** (n = 20). Regression coefficient and level of significance for the linear regression are as indicated. **(D)** Processing of DAS28 during the study was analyzed in the patient cohorts with high and low spontaneous monocyte apoptosis respectively. Change in DAS28 was calculated for the treatment time points 4, 8 12, 16 and 24 weeks. Depicted are median and standard error of the mean of numeric improvement in DAS28 (ΔDAS28) in the patient group with high (solid squares) and low (solid circles) rates of spontaneous *in vitro* SIA at baseline (BL) at indicated time points (n = 20). For significant differences between ΔDAS28 in both groups at different time points, significance levels are indicted: ^*^P <0.05, ^**^P <0.01. **(E)** Dot plot depicts the rate of spontaneous *in vitro* SIA in patients achieving a good therapeutic response after 12 weeks of etanercept (responder, Resp) (n = 20) and in patients with only moderate or no response (nonresponders, Nonresp). Significant differences are as indicated.

At baseline, no influence on the DAS28 or of individual parameters of disease activity (swollen or tender joint count, visual analog scale (VAS) or acute-phase reactants) on the rate of spontaneous monocyte apoptosis was detectable (Table 1). The baseline percentage of spontaneously apoptotic monocytes did closely correlate, however, with the reduction in DAS28 observed during the initial 12 weeks of therapy (Figure 2A). Similar results were obtained for the change in C-reactive protein (CRP) and erythrocyte sedimentation rate (ESR) values over 12 weeks (Figure 2B,C). When patients were separated into a group with high SIA comparable to healthy donors, and a group with diminished SIA, a clear separation in the clinical response became apparent. Patients with diminished monocyte SIA did not respond to treatment with a reduction in DAS28, and had significantly less reduction in DAS28 in comparison to the group with high spontaneous apoptosis after 8 weeks and at all subsequent time points (Figure 2D). Consequently, patients with a good clinical response according to the EULAR criteria had a higher rate of monocyte SIA at baseline than patients with moderate response or no response (Figure 2E).

### tmTNF reverse signaling induces secretion of IL-1sRI and IL-1sRII *in vitro* only in monocytes susceptible to high SIA

**Figure 3:**
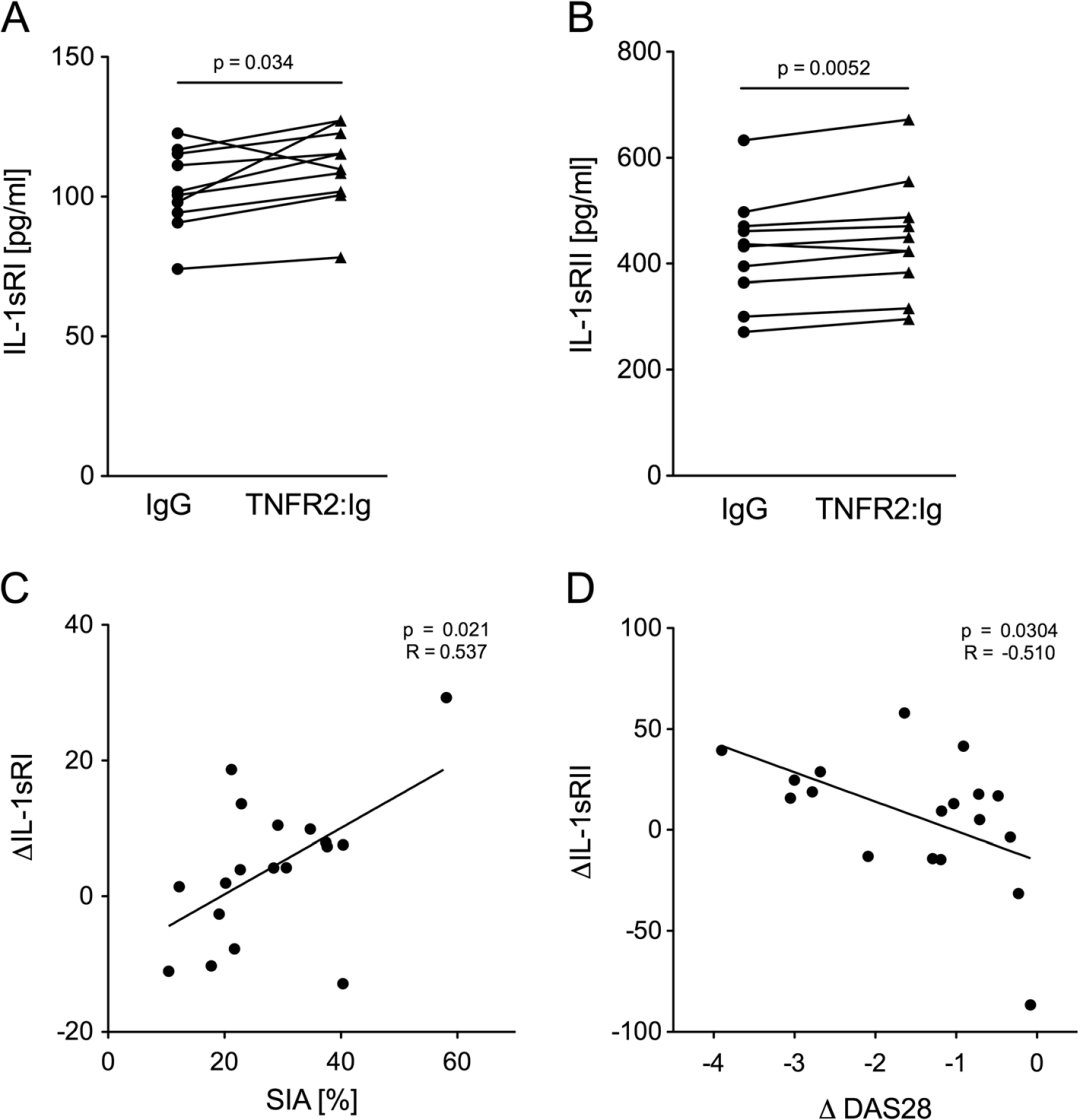
**Transmembrane (tm)TNF reverse signaling (RS)-induced secretion of soluble IL1-RI and IL1-RII depends on apoptotic rates and influences clinical response. (A, B)** tmTNF RS-induced IL-1RI and IL-1RII expression was measured in *in vitro* monocyte cultures from rheumatoid arthritis (RA) patients after baseline incubation IgG and TNFR2:Ig for 16 hours. Individually paired data of IL1sRI **(A)** and IL1sRII **(B)** concentrations in control cultures (IgG) (solid circles) and in cultures with TNFR2:Ig (solid triangles) determined *in vitro* at baseline in monocytes from the subgroup of RA patients (n = 10) characterized by high rates of spontaneous *in vitro* apoptosis (SIA) are as indicated. Significant differences are indicated. **(C)** Levels of tmTNF RS-induced IL-1RI production (ΔIL-1sRI) by RA monocytes at baseline correlates with spontaneous monocyte apoptosis in these RA patients. Dot plots depicts rate of SIA in relation to the baseline concentration of tmTNF RS-induced IL1sRI (n = 18). ∆IL-1sRI is the difference of tmTNF RS-induced IL-1sRI production and spontaneous IL-1sRI production. Regression coefficient and significane level for linear regression are indicated. **(D)** Levels of baseline tmTNF RS-induced IL-1RII production by RA monocytes correlated negatively with change (Δ) in disease activity score (DAS)28 12 weeks after starting treatment. Dot plots depicts numeric improvement in (ΔDAS28) in RA patients (n = 18) after 12 weeks of TNF blockade in relation to the concentration of tmTNF RS-induced IL1-sRII (ΔIL-1sRII) at baseline. ∆IL-1sRII is the difference in tmTNF RS-induced IL-1sRII production and spontaneous IL-1sRII production. Regression coefficient and level of significance for the linear regression are as indicated.

We have shown previously, that RS after ligation of tmTNF by anti-TNF inhibits constitutive NF-kB activation and IL-1β secretion, which subsequently increases *in vitro* apoptosis of monocytes, and which might also contribute to the therapeutic efficacy of TNF inhibitors [5]. Therefore, to investigate consequences of tmTNF RS in the present study, a wider approach was taken by determining concentrations of IL-1α, IL-1β, IL-1sRI and IL-1sRII in the supernatant of cultures with TNFR2: Ig using a cytometric bead array. No significant effect of tmTNF RS on the secretion of IL-1α or IL-1β was detectable (data not shown). Secretion of both IL-1sRI and IL-1sRII, however, was found to increase significantly following tmTNF RS, but only in monocytes with high SIA (Figure 3A and B). No increase of IL-1sRI and IL-1sRII was detectable in monocytes with diminished SIA (data not shown). Furthermore, SIA was found to be linked to tmTNF RS-induced IL-1sRI secretion of the monocytes, as a positive correlation between IL-1sRI concentrations and the rate of spontaneous apoptosis became apparent (Figure 3C). Spontaneous secretion of IL-1α and IL-1β in contrast, did not differ between patients with high or low spontaneous apoptosis.

For IL-1sRII, a significant negative correlation of the concentrations induced by tmTNF RS with the reduction in the DAS28 after 12 weeks was detected (Figure 3D), whereas a similar trend for IL-1sRI did not reach statistical significance (*R* = -0.333; *P* = 0.177).

### High susceptibility of monocytes to tmTNF RSA at baseline is associated with insufficient therapeutic response

**Figure 4:**
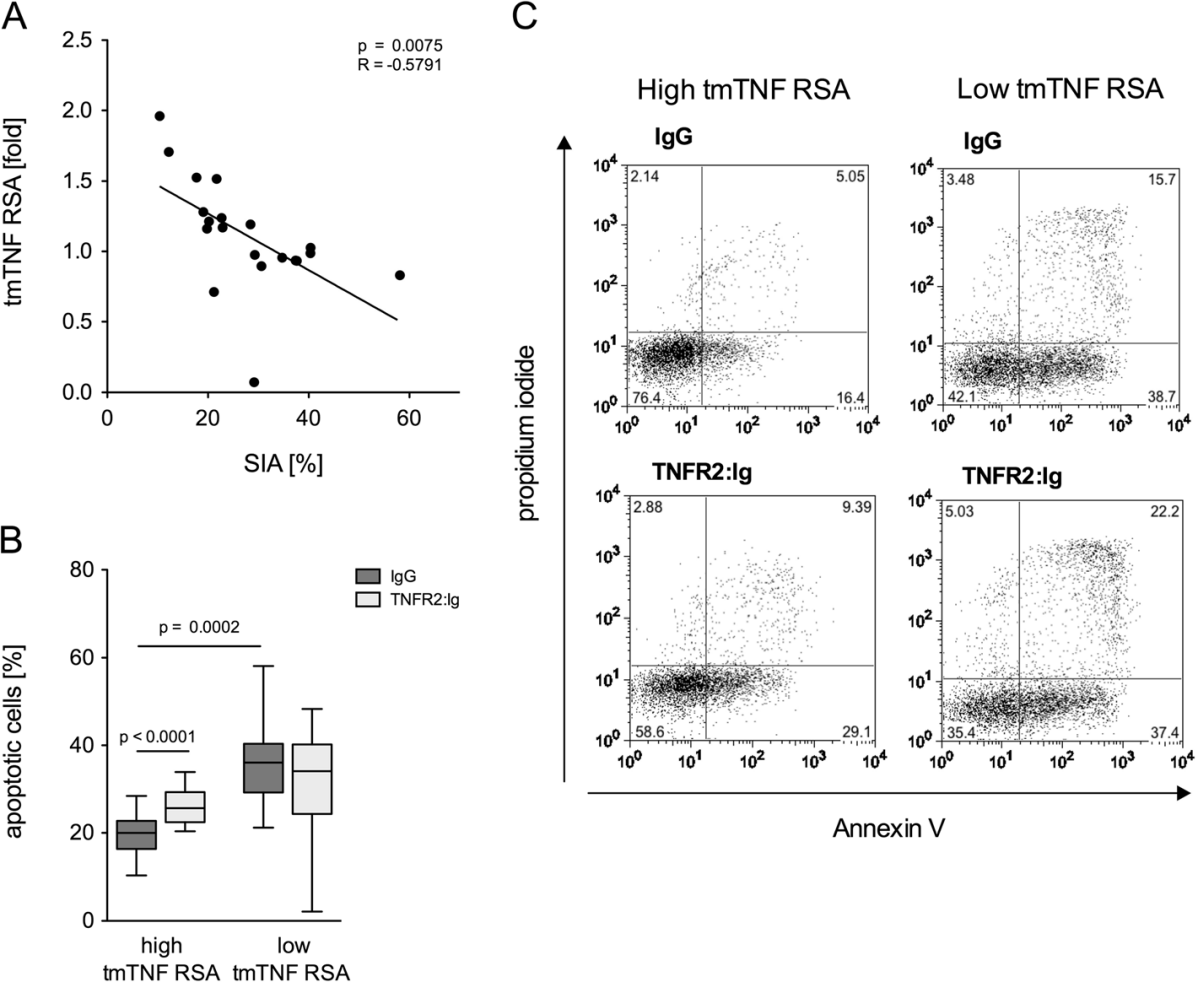
**Monocytes with low spontaneous *in vitr*o apoptosis are susceptible for transmembrane TNF reverse signaling-induced apoptosis (tmTNF RSA). (A)** Dot plot shows the rate of spontaneous *in vitro* apoptosis (SIA) in relation to tmTNF RSA) in rheumatoid arthritis (RA) patients (n = 20). tmTNF RSA is shown as ratio of TNFR2:Ig apoptosis and SIA. Regression coefficient and level of significance for the linear regression are as indicated. **(B)** Box plot depicts the rate of tmTNF RSA (light gray bars, TNFR2:Ig) in comparison to SIA (dark gray bars, IgG) in the groups with high and low tmTNF RSA separately (n = 20). Significant differences are as indicated. **(C)** Representative dot plots of annexin V- and propidium iodide-stained monocytes from one patient with high tmTNF RSA (left panels) and one with low TNF RSA (right panels). Upper panels show SIA (IgG), lower panels TNF RSA (TNFR2:Ig).

In addition to tmTNF RS-induced production of IL-1α, IL-1β and the two receptors, we also measured tmTNF RSA. In accordance with the results from previous studies [5], significant rates of RSA were only observed in monocytes with upregulated tmTNF expression (data not shown). Parallel analysis of SIA and of tmTNF RSA at baseline showed a highly significant inverse correlation between the two parameters (*P* = 0.0075, *R* = -0.5791, Figure 4A,B). When the patient cohort was separated into one group with low susceptiblity to tmTNF RSA and one with high rates of RSA, the two groups were found to differ also in their spontaneous apoptotic rates (Figure 4C).

**Figure 5:**
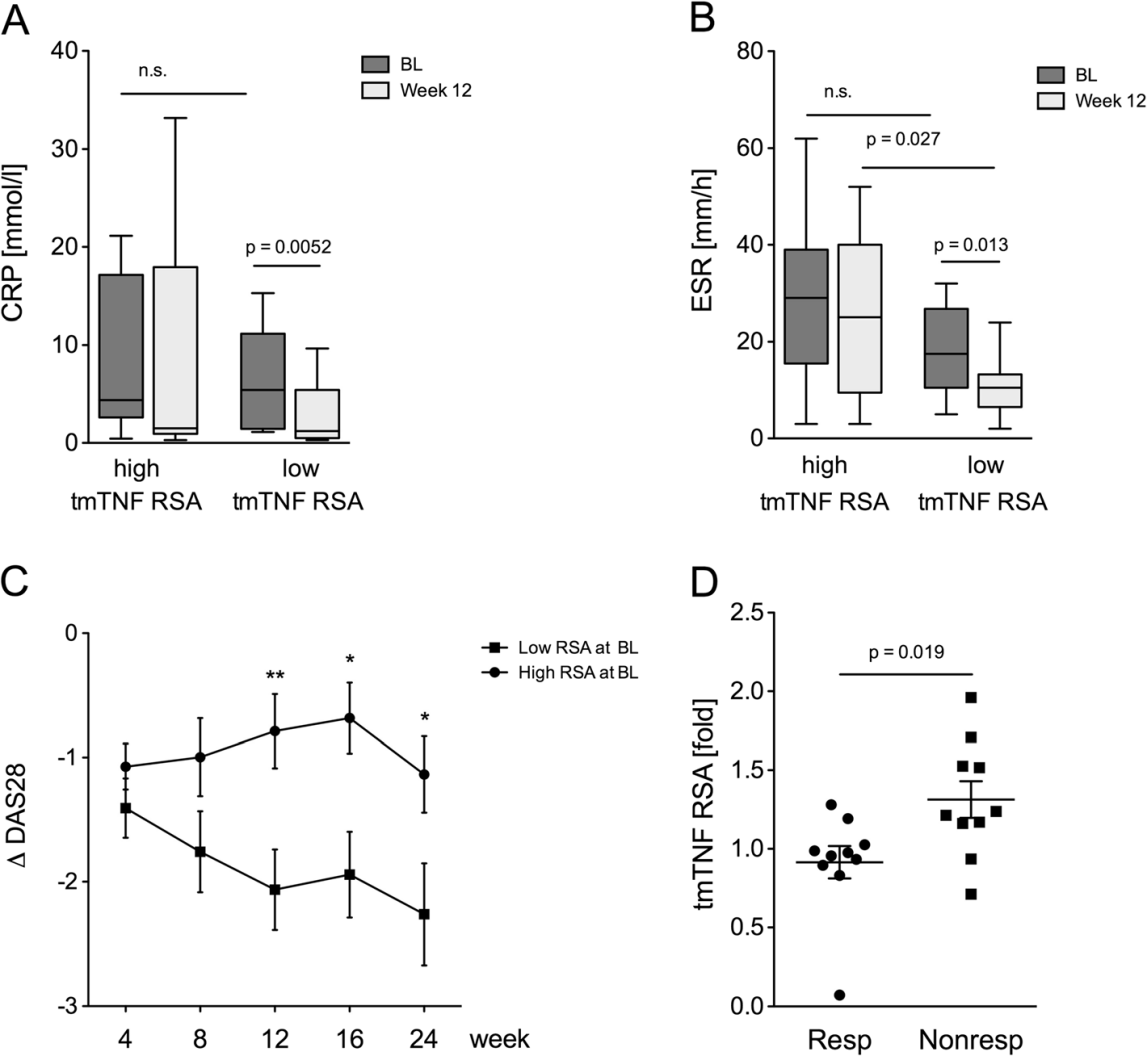
**Transmembrane (tm)TNF reverse signaling induced apoptosis of monocytes correlates with change in disease activity after 12 weeks. (A, B)** Box plots depict C-reactive protein (CRP) **(A)** and erythrocyte sedimentation rate (ESR) **(B)** at baseline (BL) (dark gray bars) and after 12 weeks of etanercept (light gray bars) in the groups with high tmTNF reverse signaling induced apoptosis (tmTNF RSA) and low tm tmTNF RSA separately (n = 20). Significant differences are as indicated. **(C)** Depicted are median and standard error of the mean of the numeric improvement (Δ) in disease activity score (DAS)28 in the patient group with high (solid squares, n = 11) and low (solid circles, n = 9) rates of tmTNF RSA at the indicated time points. For significant differences between ΔDAS28 in both groups at the different time points, the levels of significance are indicated: ^*^P <0.05, ^**^P <0.01). **(D)** Dot plot depicts the rate of tmTNF RSA in patients achieving a good therapeutic response after 12 weeks of etanercept (responder, Resp) and in patients with only a moderate or no response (nonresponders, Nonresp). Significant differences are as indicated.

The clinical analysis showed that only RA patients in the low tmTNF RSA group responded with a significant decrease of CRP (Figure 5A) and ESR (Figure 5B) during the initial 12 weeks of therapeutic TNF blockade, whereas the high tmTNF RSA group did not. Accordingly, the reduction in DAS28 (ΔDAS28) was significantly higher at all time points in the group with low tmTNF RSA at baseline (Figure 5C), and such resistance to tmTNF RSA at baseline was associated with a good clinical response to TNF blockade after 12 weeks according to the EULAR criteria (Figure 5D). Susceptibility to tmTNF RSA at baseline, in contrast, was a predictor for only moderate response or no response at all (Figure 5D).

## Discussion

The goal of our study was the investigation of spontaneous and tmTNF RS-induced monocyte apoptosis in RA patients before the initiation of therapeutic TNF blockade, followed by a longitudinal analysis of the clinical response. In this longitudinal analysis, a significant influence of decreased SIA on the clinical response to anti-TNF was revealed. The SIA of monocytes from patients with a good clinical response was higher than from nonresponders and comparable to the healthy controls in the pre-study investigation. Deficient SIA, on the other hand, was predictive of an insufficient therapeutic response, suggesting that monocyte apoptosis might also be involved in therapeutic response to TNF blockade. As the patients were not initiated on any conventional DMARD therapy in the study, we can only speculate upon the contribution of SIA towards methotrexate (MTX) response.

Resistance to *in vitro* apoptosis has been described to occur as a consequence of activation of human monocytes [15]. As several signs of activation of monocytes in RA have been described [16,17], this mechanism could indeed contribute to the observed decrease of SIA in RA patients. Accordingly, we have reported previously that the deficient SIA of RA monocytes is partly due to increased spontaneous IL-1β secretion and constitutively activated NF-kB signaling [5]. Therefore, activation of circulating monocytes is a likely cause for the deficient SIA in half of the study cohort, and might also contribute to the unfavorable therapeutic outcome in those patients. In addition, overexpression of anti-apoptotic molecules such as FLIP [7] or self-sustained NF-kB activation [18] have been described in RA, which could further reinforce resistance to apoptosis. An alternative hypothesis would be the existence of an RA-specific apoptotic defect, which aggravates the disease due to survival of pro-inflammatory monocytes.

tmTNF RS induces several effects in RA monocytes. One previously unknown finding of our study was the secretion of both type I IL-1sR (IL-1sRI) and type II IL-1sR (IL-1sRII) into the supernatant of monocytes following TNFR2:Ig-triggered tmTNF RS. This is in contrast to a previous publication reporting that treatment with TNFR2:Ig has no influence on the expression of cell surface IL-1RII *in vivo*[19], but circulating or local levels of IL-1sRII had not been determined in that study.

IL-1sRII is known to bind IL-1β with higher avidity than IL-1sRI, while not interfering with the binding of IL-1 receptor antagonist (IL-1RA) [20]. IL-1sRII has also been reported to be expressed in higher concentrations in the synovial fluid of RA patients [20]. Importantly, the simultaneous addition of IL-1sRII and IL-1RA to cultures of synovial cells leads to a synergistic inhibitory effect on the secretion of interstitial collagenase and prostaglandine E2 (PGE2) [21], indicating a potentially relevant role for IL-1sRII in the rheumatoid synovium *in vivo*. In an analogous conclusion, shedded IL-1sRII has also been suggested to act locally by dampening colonic inflammation in Crohn’s disease [22].

The essential role of the IL-1β-IL1-R system for the RA-specific resistance of monocytes against SIA was confirmed in the present study, when a positive correlation between the tmTNF RS induced IL-1sRI secretion of monocytes and their spontaneous apoptosis became apparent. This correlation indicates that the ability of cells to respond to tmTNF RS with production of IL1sRI is linked to their susceptibility to spontaneous apoptosis. One possible explanation is, that the cells prone to spontaneous apoptosis are also the ones shedding IL-1sRI upon triggering of tmTNF RS. Indeed, high concentrations of IL-1sRI and IL-1sRII were detectable only in monocytes with high spontaneous apoptosis (that is, in monocytes from patients with a good therapeutic response), which indicates that IL-1R secretion might also be beneficial *in vivo*. The close correlation between IL-1sRII and the decrease in the DAS28 indicates that IL1R secretion might also be therapeutically relevant.

The observed negative correlation between SIA and tmTNF RSA indicates that the latter only occurs in patients with a pathologic resistance to spontaneous apoptosis. In healthy controls, tmTNF RSA cannot be triggered [5], and patients with low tmTNF RSA were also the ones with a good clinical response to anti-TNF therapy. Nevertheless, this lack of tmTNF RSA in anti-TNF responders *in vitro* does not exclude a contribution of tmTNF RSA to the clinical efficacy of TNF blockade *in vivo*. In the synovial membrane, TNF blockade has indeed been linked to monocyte/macrophage apoptosis [23], although other studies detected no immediate monocyte apoptosis in the peripheral blood or the rheumatoid synovium following anti-TNF antibody infusion [24,25]. The latter would argue against a therapeutic effect of tmTNF RSA, which might instead be an indicator for the pathological state of activation of monocytes in RA. Similarly, RA monocytes are also characterized by expression of tmTNF, which correlates with the rate of tmTNF RSA, whereas monocytes from healthy donors do not express tmTNF on their cell surface [5]. Accordingly, the lack of tmTNF RSA in anti-TNF responders could be the result of a lack of tmTNF expression in those patients, which in turn might be an indicator of less severe disease.

Finally, tmTNF RS could also be part of a pro-inflammatory pathway, which is aggravating the course of RA, possibly during direct cell-cell contact [9]. Consequently, the non-responders to therapeutic TNF blockade could be the patients in whom those pro-inflammatory effects of tmTNF ligation by therapeutic anti-TNF compounds outweigh the beneficial effects of TNF blockade. Accordingly, RS in non-responders can induce apoptosis *in vitro*, but might still be deleterious to the patient due to those pro-inflammatory effects. Consequently, high rates of tmTNF RSA predict poor therapeutic response.

## Conclusion

**Figure 6:**
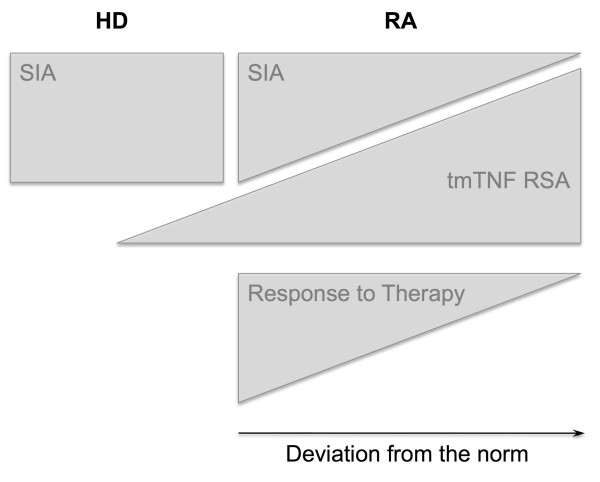
**Schematic representation of the magnitude of spontaneous *in vitro *apoptosis (SIA) and transmembrane TNF reverse signaling-induced apoptosis (tmTNF RSA) in healthy donors (HD) and patients with rheumatoid arthritis (RA).** A shift from SIA towards tmTNF RSA is associated with both disease severity and the clinical response under anti-TNF treatment.

In conclusion, we have shown that both deficient SIA and high tmTNF RSA are associated with an unfavorable outcome of TNF blockade. Ultimately, it is unclear whether resistance to SIA or increased tmTNF RSA is the primary predictor of an insufficient response, because both parameters are closely related. The findings indicate that those deviations from the pattern seen in healthy controls (which is characterized by high SIA and resistance to tmTNF RSA) are not only hallmarks of RA per se, but are also predictors of treatment failures (Figure 6). Consequently, apoptotic pathways are likely to be involved in both the pathogenesis of the disease and the therapeutic response to TNF blockade in RA.

## Abbreviations

CCP: Cyclic citrullinated peptide; CRP: C-reactive protein; DAS: Disease activity score; DMARD: Disease-modifying anti-rheumatic drug; ESR: Erythrocyte sedimentation rate; EULAR: European League Against Rheumatism; IL: Interleukin; IL-1R: Interleukin 1 receptor; IL-1RA: Interleukin 1 receptor antagonist; NSAID: Non-steroidal anti-inflammatory drug; PGE: Prostaglandin; PI: Propidium iodide; RA: Rheumatoid arthritis; RF: IgM rheumatoid factor IgM; RS: Reverse signaling; RSA: Reverse signaling-induced apoptosis; SIA: Spontaneous induced apoptosis; tmTNF: Transmembrane tumor necrosis factor; tmTNF RSA: tmTNF-reverse signaling-induced apoptosis; TNF: Tumor necrosis factor; TNFR2: Tumor necrosis factor receptor 2; TNFR2: Ig tumor necrosis factor receptor 2:immunoglobulin fusion protein; VAS: Visual analog scale.

## Competing interests

The authors declare that they have no competing interests.

## Authors’ contributions

MK und UM performed the experiments, analysis and data collection. UM, MR, MK and UW carried out data collection and statistical analysis. MK, CB and UW have done clinical evaluation of patients and sample collection. UM, MR, CB and UW participated in the experimental and study design. UM, MR and UW have written this article. All authors read and approved the final manuscript.

## Authors’ information

Undine Meusch and Maria Klinger share the first authorship. Manuela Rossol and Ulf Wagner share the senior authorship.
